# Lipid Profile Paradox: Investigating Improved Lipid Levels in Diabetic Mellitus Patients with Foot Ulcer Infections—A Prospective Descriptive Study

**DOI:** 10.3390/diagnostics13233531

**Published:** 2023-11-25

**Authors:** Andrei Ardelean, Andreea-Adriana Neamtu, Diana-Federica Balta, Carmen Neamtu, Dan Goldis, Mihai Rosu, Alexandru Nesiu, Silviu Moldovan, Cristi Tarta, Bogdan Dan Totolici

**Affiliations:** 1 Clinical County Emergency Hospital of Arad, Andrenyi Karoly Str, Nr. 2-4, 310037 Arad, Romania; andreiardelean1986@gmail.com (A.A.); aneamtu94@gmail.com (A.-A.N.); neamtu.carmen@uvvg.ro (C.N.); goldisdan@yahoo.com (D.G.); mihai.roshu@yahoo.com (M.R.); alexnesiu@yahoo.com (A.N.); totolici.bogdan@uvvg.ro (B.D.T.); 2 Faculty of Medicine, “Vasile Goldis” Western University of Arad, Liviu Rebreanu Str., Nr. 86, 310045 Arad, Romania; silviu602@gmail.com; 3 Research Center for Pharmaco-Toxicological Evaluations, “Victor Babeș” University of Medicine and Pharmacy from Timișoara, Eftimie Murgu Sq., Nr. 2, 300041 Timișoara, Romania; 4 Department X, 2nd Surgical Clinic, Researching Future Chirurgie 2, “Victor Babeș” University of Medicine and Pharmacy Timișoara, Eftimie Murgu Sq., Nr. 2, 300041 Timișoara, Romania

**Keywords:** type 2 diabetes mellitus, infected diabetic foot ulcers, lipid profile, total cholesterol, HDL-C, LDL-C, ApoE, triglycerides

## Abstract

Type 2 diabetes mellitus (DM) is a chronic metabolic disorder posing multifaceted challenges to global public health. Among its numerous complications, infected diabetic foot ulcers (IDFUs) represent a particularly debilitating consequence. Beyond cardiovascular implications, there is an emerging understanding of the interconnectedness among IDFUs, neuropathy, atherosclerosis, and dyslipidemia. IDFUs, peripheral neuropathy, and atherosclerosis share common risk factors and mechanistic pathways. The primary objective of this study was to characterize the lipid profiles in DM patients with IDFUs, comparing them with DM patients without foot ulcers, and with a control group of healthy subjects. The secondary objectives included evaluating apolipoprotein E (ApoE) levels across these study groups and comparing lipid profiles within IDFU subgroups. A total of 160 patients were assessed for this study. After applying exclusion criteria, 140 participants were included, divided into three groups: Group IDFU (80 patients with IDFUs), Group DM (32 patients with DM but no foot ulcers), and Group Controls (28 healthy controls). Compared to Group DM, Group IDFU demonstrated lower levels of high-density lipoprotein cholesterol (HDL-C) (30.9 ± 12.6 mg/dL vs. 40.8 ± 16.6 mg/dL, *p* = 0.002), but improved levels of ApoE (160.9 ± 68.4 mg/dL vs. 197.2 ± 69.6 mg/dL, *p* = 0.01), triglycerides (TG) (126.9 ± 56.2 mg/dL vs. 165.8 ± 79.0 mg/dL, *p* = 0.004), low-density lipoprotein cholesterol (LDL-C) (84.2 ± 32.3 mg/dL vs. 92.3 ± 39.3 mg/dL, *p* = 0.1), and total cholesterol (133.6 ± 43 mg/dL vs. 164.6 ± 44.4 mg/dL, *p* = 0.002). The IDFU patients exhibit improved lipid profiles, excepting HDL-C, which is unusual because IDFU follows complications related to dyslipidemia for DM patients. Anemia, impaired renal function, and elevated TG levels were identified as biomarkers for mortality among patients with IDFUs. The data suggest that a lower level of HDL-C and an improved lipid profile may indicate a systemic end-stage disease manifestation in DM patients with IDFUs.

## 1. Introduction

Type 2 diabetes mellitus (DM), a chronic metabolic disorder, presents multifaceted challenges to global public health. Among its myriad complications, the diabetic foot ulcer (DFU) stands out as a particularly debilitating consequence. Not only does it impair the quality of life for those affected, but it also poses significant economic challenges, both to affected individuals and healthcare systems worldwide [1].

The stark reality is that the DFU is more than just a medical condition; it represents a comprehensive burden encompassing the spheres of physical, emotional, and financial strain [2]. Approximately 15% of all individuals with diabetes mellitus will develop a foot ulcer at some point in their lifetime. Moreover, DFUs precede 85% of diabetes-related amputations, leading to a cascade of downstream consequences, both medical and socio-economic [3]. A growing body of literature suggests that the presence of a diabetic foot ulcer is an independent risk factor for mortality. Brownrigg et al. reported that foot ulceration significantly influenced cause-specific mortality in diabetic patients; patients with IDFUs died three years earlier than DM patients with no ulcer, drawing attention to the systemic implications of a localized foot disease [4]. In a population-based cohort study from the United Kingdom, it was observed that diabetic foot ulcers were associated with an elevated risk of death, emphasizing the life-threatening nature of this common diabetic complication [5]. Furthermore, the independent contribution of DFUs on lower extremity amputation and increased mortality risk were shown to lead the patient onto the often-irreversible trajectory from ulceration to severe outcomes [6].

The link between DFUs and mortality could also be explained by the association with cardiovascular events. Dietrich et al. described the diabetic foot as a “proxy” for cardiovascular events and mortality, pointing out a significant correlation between the presence of foot ulcers and the likelihood of adverse cardiovascular outcomes [7]. Long-term data paint a similarly grim picture, with over a decade of data revealing that the rates of amputation and death among diabetic foot patients are disturbingly high—45% at 5-year follow-up, respectively, and 70% at 10-year follow-up—reinforcing the need for prevention, aggressive management, and the development of alternative treatment strategies [8,9]. 

Armstrong et al. compare the mortality rates at five years of diabetic wounds and amputations to different types of cancer, suggesting that their impact may be as severe, or even worse, in terms of mortality rates: diabetic foot ulcers were equal to colonic cancers with a five-year death rate, ischemic ulcers surpassed the colonic cancers, and ulcers and the need for revascularization were the third most deadly factors for patients, after lung and pancreatic cancers [10]. The recurrent nature of DFUs further compounds the problem, as each new ulcer episode increases the risk of adverse outcomes including mortality, in addition to the healthcare costs associated to the progressive disease [11].

As healthcare costs continue to escalate, the economic burden of DFUs cannot be overlooked. An analysis by Driver et al. makes a compelling argument for the creation of specialized limb salvage teams by highlighting the staggering costs associated with the care of diabetic foot complications. Hospitalizations, prolonged wound care, surgeries, and the costs associated with rehabilitation are just the tip of the iceberg. Lost work hours, decreased productivity, and the psychological impact on affected individuals further add to the economic implications [12]. Considering the aforementioned context, it is evident that DFUs are a matter of global concern that require rigorous study and dedicated resources. 

Diabetic dyslipidemia is a frequent lipoprotein metabolism disturbance observed in patients with type 2 diabetes mellitus. This condition is marked by a triad of elevated triglycerides (TGs), diminished high-density lipoprotein cholesterol (HDL-C) levels, and the prevalence of small, dense, low-density lipoprotein (sdLDL) particles [13,14]. Even in DM patients who maintain optimal metabolic control, while the concentration of LDL-cholesterol (LDL-C) may remain within the normal range, significant qualitative alterations in LDL-C particles can still be discerned [13,15]. Intriguingly, sdLDL has recently been identified as a major risk factor for the initiation of cardiovascular disease (CVD) in patients with DM [15]. 

Beyond the cardiovascular implications, there is emerging recognition of the interrelation between DFU, neuropathy, atherosclerosis, and dyslipidemia. DFU, peripheric neuropathy, and atherosclerosis exhibit shared risk factors and mechanistic pathways [7,16,17]. This overlapping nature hints at the potential influence of dyslipidemia in the onset and progression of these complications. However, while the link between DM’s cardiovascular complications and dyslipidemia has been extensively researched, data on the role of lipid biomarkers in DFUs remain comparatively sparse, underscoring a need for further investigation in this area. 

Existing evidence implies that standard lipid profile indicators, notably, TG and HDL-C levels, could be instrumental in evaluating the risk of DFU development [18]. The predictive capacity of the lipid biomarkers, however, is yet to be confirmed. While the exact mechanisms tying atherogenic lipoproteins to DFU development are not fully understood, a synergy of dyslipidemia, hyperglycemia, oxidative stress, and inflammation might set the stage for the exacerbation of DFU-related issues [17,19]. A crucial benefit of consistent lipid biomarker screening could be pinpointing DFU patients who stand to gain the most from cutting-edge diabetes treatments. These treatments have the potential to substantially decrease atherogenic lipoprotein levels, thereby potentially averting or postponing foot-related complications [14].

Apolipoprotein-E (ApoE) is a central component of plasma lipoproteins involved in lipid transport among cells across different organs and specific tissues. It is associated with various lipoproteins and plays a crucial role in clearing these lipoproteins from the plasma by binding them to specific cell-surface receptors such as the LDL-C receptor family [20,21]. ApoE is essential in the maintenance of plasma cholesterol homeostasis, assisting in hepatic uptake of lipoproteins, and other functions including cholesterol efflux stimulation from macrophages, and the prevention of platelet aggregation. Its protective role against atherosclerosis is significant, with diminished ApoE expression linked to a proatherogenic lipoprotein profile and advanced atherosclerotic disease [22]. While ApoE is typically absent in normal vessels, its expression surges in atherosclerotic plaques, predominantly synthesized by local macrophages. Studies confirm the anti-atherogenic function of macrophage-derived ApoE, indicating its critical role in lipid homeostasis and atherosclerosis management [23,24,25]. 

The objective of this study is to characterize lipid profiles of DM patients with IDFUs and DM patients with no DFU, and compare them with a group of healthy subjects. The secondary objectives are to evaluate the ApoE levels of these groups, and to compare lipid profiles among subgroups of IDFU patients. 

## 2. Materials and Methods

### 2.1. Study Participants and Group Formation

A single-site prospective study was carried out in a tertiary hospital, the Clinical County Emergency Hospital of Arad, in the period March 2020–March 2022. Participating individuals were briefed about the intent of the study, and granted their informed consent before joining.

Three distinct patient groups were established:

- Group IDFU—90 individuals with IDFU:
  ○ Further divided based on the level of surgery (distal or proximal) and the outcome at the six-month follow-up (survivors or non-survivors):
    ▪ Distal level (below the ankle) surgical procedure subgroup:
      1. Surgical debridement without bone amputation;
      2. Toe removal;
      3. Transmetatarsal removal;
      4. Midtarsal removal;
    ▪ Proximal level (above the ankle) surgical procedure subgroup:
      5. Below-knee removal;
      6. Above-knee removal.
    ● Survivors;
    ● Non-survivors.
- Group DM—40 individuals with DM, but no DFU;
- Group Controls, reference control group—30 healthy individuals without inflammatory conditions, matched in terms of age and gender with the prior groups, selected from the people who came to the hospital for various regular check-ups required by their profession.

The IWGDF 2019 guidelines were utilized to categorize IDFU patients [26]. 

### 2.2. Inclusion and Exclusion Criteria

In order to be included in the study, participants had to:

1. be over 18 years old;
2. have DM type 2;
3. comprehend and give informed consent;
4. have an IDFU without prior medical or surgical treatment;
5. fall under the IWGDF’s mild-to-moderate IDFU bracket;
6. test positive for wound microbiology.

Exclusion criteria aimed at bias reduction focused on:

1. presence of other infections;
2. death from COVID-19;
3. simultaneous inflammatory diseases in the control group;
4. diagnosed malignancy, untraceable after six months or requiring major vascular surgeries.

For the DM and control group, exclusion criteria were:

1. limb vascular surgery;
2. diagnosis of DFU before the 6-month follow-up;
3. newly diagnosed DM for controls.

### 2.3. Ethical Considerations

Approval number 51st, from 24 February 2020, was acquired from the Institutional Review Board and Ethics Committee of the Clinical County Emergency Hospital of Arad, Romania. The study abided by the Declaration of Helsinki. All participants provided consent for data collection, analysis, and publication in an anonymous format.

### 2.4. Patient Characteristics

Demographics, health metrics, infection localization, amputation details, and hospitalization duration were extracted from patient records. Critical decisions, such as amputation, emerged from comprehensive daily evaluations of each individual patient. Routine blood tests were performed and documented upon admission, including the extraction of the lipid profile. ApoE values were measured using the kits and instructions of the manufacturer for APOE (Human) ELISA Kit, Abnova (Taipei, Taiwan). 

### 2.5. Concluding Follow-Up Visit

The concluding follow-up visit was scheduled for six months post-admission. This incorporated an outpatient clinic visit, with any subsequent hospital visits or procedures recorded. The DM group was questioned about the debut or the healing of a DFU in the period between inclusion in the study and FU. Mortality data were gathered from the local death registry. 

### 2.6. Data Analysis

Statistical evaluation was conducted using the software MedCalc 15.0 version. We assessed the distribution of numerical data using the Kolmogorov–Smirnov test. For data following a Gaussian distribution, we presented results as mean value alongside the standard deviation or error. Conversely, non-Gaussian data were portrayed using median values alongside the respective range interval. The *t*-test facilitated the comparison of continuous, Gaussian-distributed variables, whereas the Mann–Whitney U test was employed for non-Gaussian variables. Categorical data comparisons utilized the Chi-square test. To determine correlations between continuous variables and gauge monotonic relationships, we applied the Pearson (r) and Spearman (rho) coefficients. A *p*-value below 0.05 was deemed statistically significant. The post-hoc statistical power of the study was calculated using the software ClinCalc for independent study groups and continuous variables following Gaussian distribution, with the type I error probability α = 0.05 and type II error probability β = 0.2 [27,28,29]. According to a systematic review, the risk of diabetic patients that develop IDFU to have dyslipidemia is approximately 1.5 compared to DM, but no DFU [18]. Therefore, a relevant study group sample size for α = 0.05 and β = 0.2 error parameters (study power of 80%) with a 3:1 enrollment ratio would be 51 patients pertaining to the IDFU group, 17 patients pertaining to the DM group, and 29 patients pertaining to the control group.

## 3. Results

**Figure 1:**
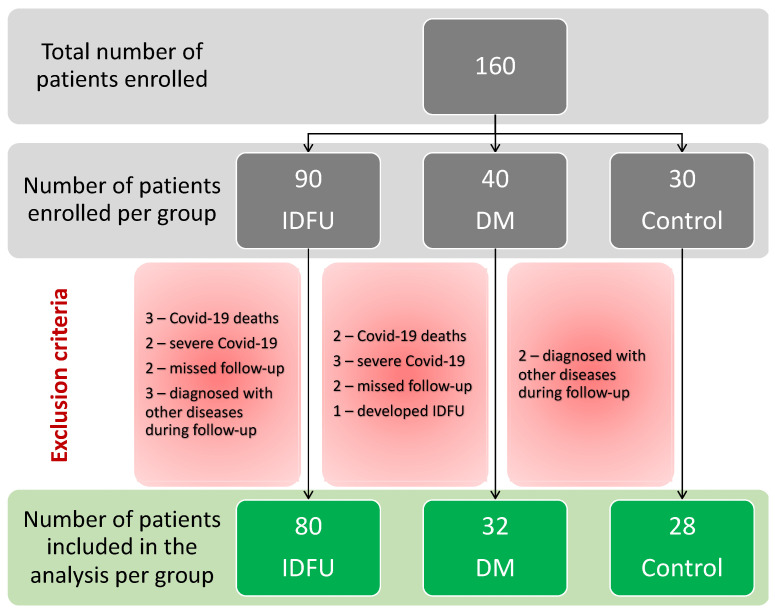
Flowchart of inclusion and exclusion of the patients in the study.

A total of 160 patients were initially enrolled in the study, with 90 patients in the IDFU group, 40 patients in the DM group, and 30 healthy controls in the Controls group. Only 140 patients were included in the data analysis after different exclusion criteria were applied (Figure 1). 

There were surprising differences in favor of group IDFU in terms of lipid profile, when comparing groups IDFU and DM (Table 1).

Furthermore, we compared group IDFU with Controls and found that the results were mixed in terms of dyslipidemia (Table 2). 

We compared the different subgroups of the IDFU group based on the type of surgical procedure and level of amputation (below or above the ankle), with patients without amputation and only debridement being allocated to the first subgroup (Table 3).

In the final table of our study, we compared the survivors with the non-survivors from the IDFU group (Table 4).

Female patients with IDFUs had a higher rate of mortality compared to men, of 19.0%, respectively, and 10.2%, at the six-month follow-up.

## 4. Discussion

Through this study, we observed that patients with infected diabetic foot ulcers had lower HDL-C levels compared with the group of patients with DM and no DFU, but, otherwise, better lipid profiles in terms of statistically significant lower total cholesterol, LDL-C, and triglycerides. 

Low levels of high-density lipoprotein cholesterol are commonly observed in individuals with metabolic syndrome and diabetes mellitus [30]. Furthermore, diminished HDL-C levels are linked to systemic inflammation and are prevalent among individuals who smoke, suffer from chronic inflammatory conditions, or have chronic kidney disease [31]. In extreme instances of low HDL-C, one should consider the possibility of rare conditions such as malignancies or an elevated susceptibility to sepsis [32]. All the above are potential scenarios for a patient with IDFUs, which typically manifests as systemic inflammation. These patients often exhibit various chronic conditions, and frequently experience some degree of renal impairment, attributed to the progression of their diabetes mellitus; moreover, they may present with sepsis.

A decrease in HDL-C levels serves as an important biomarker, prompting further investigation into underlying metabolic and inflammatory conditions. HDL-C plays multiple roles, including participating in reverse cholesterol transport, as well as exhibiting anti-oxidative and anti-inflammatory properties [33,34]. HDL-C also contributes to endothelial function by preventing the inappropriate adhesion of monocytes to the endothelial lining and aiding in endothelial repair [34,35]. A composition analysis of HDL-C has revealed the presence of enzymes, acute-phase proteins, components of the complement cascade, and protease inhibitors, in addition to the signature apolipoproteins [36].

Moreover, HDL-C has demonstrated antithrombotic and profibrinolytic activities [37,38]. It has been shown to guard against cellular damage, necrosis, and apoptosis, including playing protective roles in pancreatic beta cells. These actions suggest a potential for HDL-C to safeguard insulin secretion and avert the onset of diabetes mellitus [39,40]. HDL-C’s roles in anti-inflammation, cytoprotection, and wound healing qualify it as an element of the innate immune system [41,42]. 

It is noteworthy that several studies indicate the vascular benefits of HDL-C may not directly correlate with circulating HDL-C levels, particularly in populations with diabetes mellitus, coronary disease, chronic kidney failure, and other cardiovascular risk factors. In such populations, HDL-C functionality appears to be compromised [43].

Lifestyle modifications that elevate HDL-C levels, such as quitting smoking and increasing physical activity, are recommended. While smoking cessation is a viable strategy for enhancing HDL-C levels, physical exercise may not be a practical approach for patients with ischemic diabetic foot ulcers (IDFUs), primarily due to their mobility limitations [44,45].

Our study recorded one of the lowest HDL-C values for patients with IDFUs, registering at 30.4 mg/dL. When compared to the most recent literature review, only two studies reported lower values [18]. However, it is worth noting that the data in this review varied widely, with some studies showing HDL-C levels exceeding 50 mg/dL [18]. Geographical factors appear to influence lipid profiles, which may account for some of the observed discrepancies [46].

Previous research has demonstrated that reduced HDL-C levels are associated with impaired wound healing in patients with diabetic foot ulcers (DFUs) [47]. Furthermore, the likelihood of lower limb amputation was found to be 2.45 times greater in patients exhibiting lower HDL-C levels [48]. In our study, the HDL-C did not reach statistical significance between proximal or distal amputation of the lower limb, neither between survivors nor deceased, but the lowest value of 25.5 mg/dL was noted in the non-survivors. 

In our study, triglyceride (TG) levels were significantly lower in patients with IDFUs compared to those with diabetes mellitus (DM) but without DFUs. However, no statistical difference was observed between the IDFU group and the control group. This finding is consistent with previous studies that have observed similar trends in populations from the Balkan region [49]. Additionally, there are existing reports that indicate lower TG levels in IDFU patients when compared to those with DM [18].

TG levels, along with HDL-C, have been identified as key risk factors for the development of diabetic foot ulcers [50]. The observed reduction in TG levels in our cohort may be attributed to the consumptive nature of the disease, which often involves pain, an altered lifestyle, and depression [51]; others found the same reduction of TG levels as a risk factor for amputation among IDFU patients [52,53]. In existing literature, TG levels among DM patients with DFUs have shown significant variation, ranging from above 600 mg/dL to within normal ranges [18]. Apart from geographical variances and patients’ selection, another possible explanation for this variation could be medication use. 

Triglycerides have been increasingly recognized as a significant factor in the pathogenesis of diabetic neuropathy, a debilitating complication of diabetes mellitus. Elevated TG levels are thought to contribute to oxidative stress and inflammation, processes that are central to nerve damage in diabetic neuropathy. Elevated TG levels have been correlated with worsening neuropathic symptoms and structural nerve abnormalities, suggesting a direct role in nerve fiber degeneration [54]. High TG levels may also indirectly exacerbate neuropathy by contributing to insulin resistance, thereby worsening glycemic control, and perpetuating a cycle of metabolic dysfunction and neural injury [55]. In our study, patients with major amputation had lower statistically significant TG levels compared to below-ankle amputation patients, whereas non-survivors showed significant increased TG levels compared to non-survivors. 

LDL-C was not different between the IDFU group and DM group. Total cholesterol was significantly lower in the IDFU group. The situation was the same with the ApoE. The conclusion is that patients with IDFUs had a “better” lipid profile, excepting HDL-C, which is under the influence of multiple inflammatory factors. This improvement of the lipid profile was observed in other chronic terminal illnesses such as end-stage renal disease and liver cirrhosis.

In patients with end-stage renal disease (ESRD), elevated cholesterol levels have paradoxically been linked with improved survival outcomes. This phenomenon, known as reverse epidemiology, is influenced by factors such as malnutrition and chronic inflammation. Dyslipidemia is frequently observed in ESRD patients, attributable to shifts in lipid metabolism and alterations in the composition of plasma lipoproteins. A conventional lipid profile in this patient group often reveals normal or reduced levels of LDL cholesterol and elevated triglycerides due to increased levels of very-low-density lipoprotein and intermediate-density lipoprotein and lowered high-density lipoprotein cholesterol [56].

While it has been hypothesized that dyslipidemia might impact cardiovascular outcomes in ESRD patients, numerous studies have been inconclusive in establishing a direct relationship between traditional lipid metrics (total cholesterol, LDL-C, HDL-C, and triglycerides) and cardiovascular endpoints. In fact, a counterintuitive pattern has been noted where lower cholesterol levels correspond with higher mortality rates. Initially thought to challenge the role of cholesterol in cardiovascular disease within this population, it is now understood that this reverse epidemiology is confounded by underlying factors such as malnutrition and chronic inflammation [57,58].

In individuals with liver cirrhosis, there is a gradual decline in lipoprotein function, which correlates with heightened morbidity and mortality rates. Reduced lipoprotein levels are commonly observed in cirrhosis patients, and multiple studies confirm that hypolipidemia holds prognostic significance in these individuals. Specifically, low cholesterol levels serve as an independent indicator for survival prospects in those with cirrhosis. Liver dysfunction leads to changes in circulating lipid profiles. The diminishing levels of lipoproteins are a critical factor affecting not just the lifespan but also the onset of specific complications in patients suffering from cirrhosis [59,60].

Both end-stage renal disease and advanced liver cirrhosis exhibit lipid profile alterations that notably parallel our findings in patients with IDFUs. This further substantiates the elevated mortality rates observed in diabetic foot ulcer patients when compared to those with diabetes mellitus but without foot ulcerations. Such observations may imply that IDFU represents a terminal-stage variant of DM. In this context, the foot ulcer serves not merely as an isolated symptom, but rather as an indicator of underlying systemic metabolic and inflammatory derangements affecting the entire organism.

One surprising finding was that IDFU patients compared to healthy controls had significantly lower LDL-C and TC; only ApoE was higher, and HDL-C was much lower. 

The survival of the patients with IDFUs was linked with anemia, renal function, and triglyceride levels. 

The relationship between anemia and the prognostic outlook of DFUs is intricate, and it significantly impacts the decisions regarding minor or major amputations, along with affecting the mortality rate of patients harboring DFUs. Anemia, particularly in diabetic patients, is often a harbinger of diminished oxygen-carrying capacity and is correlated with poor wound healing owing to impaired oxygen delivery to the tissues [61].

Research suggests that anemic patients with DFUs are more prone to adverse outcomes such as infections, gangrene, and subsequent amputations, compared to their non-anemic counterparts. A systematic evaluation of hematological parameters in these patients often reveals decreased hemoglobin levels, a marker and potential mediator of increased morbidity and mortality [62]. 

The concurrence of anemia and DFUs augments the risk of minor or major amputations, with hypoxia-inducible factors having pivotal roles in tissue hypoxia, leading to reduced cellular proliferation, impaired angiogenesis, and suboptimal extracellular matrix deposition [63]. 

Moreover, the intertwined etiologies of anemia and diabetes contribute to a composite exacerbation of systemic inflammation and oxidative stress, consequently perpetuating the progression of DFUs and compromising the therapeutic prospects. The association between reduced hemoglobin levels and elevated mortality in DFU patients accentuates the need for prompt, rigorous intervention strategies and optimal glycemic control to ameliorate the potential ramifications on patient survival [64]. 

As in our study, IDFU patients showed more instances of anemia compared to the DM group; anemia was also associated with major limb amputation (*p* = 0.02) and with mortality (*p* = 0.005), the same results as those found in different studies around the world [53,65].

The female sex was a bad prognostic feature for survival in our study with a double percentage compared to men; this could be due to the short follow-up period of only six months or a geographical variable. IDFUs manifest significant gender-based discrepancies in their prognostic outcomes, particularly concerning amputation and mortality. Men with DFUs are demonstrated to incur a roughly 50% heightened risk for amputation compared to women [66], with underlying mechanisms remaining partially obscure. These divergences might be attributed to the variations in healthcare accessibility, utilization patterns, and perceived criticality of illnesses between genders, leading to delayed or inadequate interventions particularly in men [67].

Furthermore, gender disparities are evident in occupational domains, exacerbating ulcer recovery times and amputation propensities in men due to higher incidences of physically strenuous activities and absenteeism [68]. Nonetheless, despite men exhibiting a higher frequency of LEAs, they are generally younger at the time of amputation and have lower associated mortality rates than women, illustrating a complex interplay between gender, DFUs, amputation risk, and mortality [69].

This emphasizes an urgent need for comprehensive, gender-sensitive research and interventions in diabetic care, aimed at attenuating disparities and enhancing outcomes, entailing meticulous management and healthcare strategies, particularly focusing on men’s health education and women’s mortality risk [67].

Apolipoprotein-E has been highlighted in various studies in connection with DM type 2 and its complications. ApoE is a glycoprotein crucial for lipid metabolism, with polymorphism in its gene potentially influencing the susceptibility to metabolic disorders and their subsequent complications, such as diabetic foot ulcers and nephropathy. Several studies examined this relationship and found a significant correlation between ApoE gene polymorphism and the presence of diabetic foot ulcers or serum lipid concentrations, implicating the gene variant in the increased risk and severity of this condition [70,71]. The intricacies of ApoE in metabolic conditions were also explored, demonstrating its correlation with diabetic peripheral neuropathy [72]. 

ApoE and its alleles have a pivotal role not only in healthy individuals but also in pathological states like atherosclerosis, further emphasizing its critical impact in various cardiovascular and metabolic conditions leading to IDFUs [73]. 

This array of research underscores the multidimensional impact of ApoE gene polymorphism, particularly in diabetic complications, accentuating the necessity for further explorative and conclusive studies to fully elucidate the implications and mechanisms underlying these associations. We found that ApoE was higher in patients with DM compared to DM patients with IDFUs, but lower in controls, even though the lipid profile was not worse in IDFU patients, excepting HDL-C.

Poor glycemic control, a hallmark of poorly managed diabetes, is identified as a major risk factor for the development of DFUs in the literature [16]. Elevated blood glucose levels can lead to complications such as peripheral neuropathy and peripheral arterial disease, which are precursors to the development of foot ulcers. Additionally, impaired immune function associated with uncontrolled diabetes can hinder the healing process of wounds, thereby increasing the likelihood of ulceration and subsequent infection [16]. We did not find this relation in our study as HbA1c was not found to be statistically significant between our groups; statistical significance was found between the above-the-ankle amputees’ group and the below-the-ankle group, with a lower HbA1c for major amputees. 

The period of the COVID-19 pandemic was fraught with health-related challenges, many stemming from infections, while others were a direct result of stress, emotional crises, and the general state of shock induced by the COVID-19 pandemic. Isolation, dietary changes due to lockdowns, and other factors have contributed significantly to these challenges. These changes have posed significant threats to individuals with diabetic foot problems, including active DFUs, ischemia, and Charcot neuroarthropathy, as a comprehensive clinical examination is paramount for the accurate assessment and management of diabetic foot disease. Moreover, the fear of hospital exposure to COVID-19 has resulted in many individuals with diabetes avoiding necessary hospital visits, further complicating the management of diabetic foot complications [74]. We excluded the patients with a positive COVID-19 test in order to lower the bias in our study regarding mortality.

Our study has several potential sources of bias: the number of the patients, which is not as high; the relatively short follow-up period of just six months; and the lack of comparison of the diabetic neuropathy. Nonetheless, none of the above could change the lipid profile of the subjects included in our study. Another source of bias was the limitation in enrolling patients with various types of treatment for type 2 DM and not assessing their adherence to these treatments due to several reasons, including social and economic factors, as well as the challenges posed by the pandemic period. This might have influenced the results of our lipid-profiling research, as previously demonstrated [75]. 

Future research in this field might bring new and more precise insights. Lipidomics, the extensive study of lipid molecules, could be pivotal in unraveling the complexities of metabolic alterations, specifically in conditions like IDFU. By comprehensively analyzing lipid profiles, lipidomics aids in discerning alterations in lipid metabolic pathways, potentially unveiling novel biomarkers and therapeutic targets for IDFUs. The intricate landscape of lipids in IDFUs is crucial, as dysregulated lipid metabolism is integrally associated with inflammation and impaired wound healing, common characteristics of IDFUs. Unraveling lipidomic profiles in IDFU patients could lead to a nuanced understanding of the metabolic shifts occurring within the wound microenvironment, potentially illuminating the pathways leading to the delayed wound healing characteristic of IDFUs. The exploration of lipid molecules, and their interactions and roles could elucidate the mechanistic insights into the development and progression of IDFUs, paving the way for targeted therapeutic strategies [76,77]. 

## 5. Conclusions

A paradoxical improvement in the lipid profile and a lower HDL-C in patients with infected diabetic foot ulcers seem to indicate a worsening progressive systemic disease, with IDFUs representing just a visible alarm signal. Anemia, impaired renal function, and elevated triglyceride levels were identified as biomarkers for mortality among patients with IDFUs. 

## Figures and Tables

**Table 1:** Demographics and lipid profiles of groups IDFU and DM.

Parameter	IDFU Group (n = 80)	DM Group (n = 32)	*p*-Value	Post-Hoc Statistical Power (α = 0.05, β = 0.2)
Male gender	67.5% (54/80)	62.5% (20/32)	0.68	-
Age [years]	64.3 ± 10.3	62.6 ± 11.0	0.54	-
Rural provenience	62.5% (50/80)	68.75% (22/32)	0.61	-
BMI kg/m^2^	26.4 ± 4.8	26.9 ± 5.1	0.82	-
Hemoglobin (Hb) [g/dL]	11.2 ± 2.1	13.1 ± 2.2	0.0002	98.7%
Hematocrit (Ht) [%]	33.9 ± 5.8	39.5 ± 5.8	0.0001	99.6%
HbA1c [%]	8.55 (4.7–16.9)	8.9 (5.6–14.6)	0.8	-
Creatinine [mg/dL]	0.98 (0.45–3.23)	0.91 (0.36–2.84)	0.45	-
Urea [mg/dL]	54.8 ± 37.0	43.9 ± 24.8	0.17	-
HDL cholesterol [mg/dL]	30.9 ± 12.6	40.8 ± 16.6	0.002	86.0%
LDL cholesterol [mg/dL]	84.2 ± 32.3	92.3 ± 39.3	0.1	-
Total cholesterol [mg/dL]	133.6 ± 43.0	164.6 ± 44.4	0.002	92.0%
Triglycerides [mg/dL]	126.9 ± 56.2	165.8 ± 79.0	0.004	71.9%
Apolipoprotein-E level [mg/dL]	16.1 ± 6.8	19.7 ± 6.9	0.01	70.7%
Diabetes duration [%] 5 years 5–10 years 10 years	26.25 35.00 38.75	34.38 40.62 25.00	0.27	-

Note: *p*-values in grey are deemed statistically significant (*p* < 0.05). BMI—body mass index.

**Table 2:** Comparison of the lipid profiles between IDFU patients and healthy controls.

Parameter	IDFU Group (n = 80)	Controls Group (n = 28)	*p*-Value	Post-Hoc Statistical Power (α = 0.05, β = 0.2)
BMI [kg/m^2^]	26.4 ± 4.8	27.2 ± 5.2	0.64	-
HDL cholesterol [mg/dL]	30.9 ± 12.6	51.0 ± 13.7	<0.0001	100%
LDL cholesterol [mg/dL]	84.2 ± 32.3	122.8 ± 29.2	<0.0001	100%
Total cholesterol [mg/dL]	133.6 ± 43.0	195.7 ± 32.7	<0.0001	100%
Triglycerides [mg/dL]	126.9 ± 56.2	115.6 ± 57.0	0.39	-
Apolipoprotein-E level [mg/dL]	16.1 ± 6.8	5.5 ± 1.3	<0.0001	100%

Note: *p*-values in grey are deemed statistically significant (*p* < 0.05).

**Table 3:** Compared lipid profiles of IDFU subgroups: below or above the ankle surgery.

Parameter	Below-the-Ankle Group (n = 63)	Above-the-Ankle Group (n = 17)	*p*-Value	Post-Hoc Statistical Power (α = 0.05, β = 0.2)
Male gender	68.2% (43/63)	64.7% (11/17)	0.90	-
Age [years]	64.7 ± 10.6	67.5 ± 10.8	0.33	-
Hemoglobin (Hb) [g/dL]	11.5 ± 2.1	10.1 ± 1.8	0.02	78.3%
Hematocrit (Ht) [%]	34.8 ± 5.8	30.7 ± 4.7	0.01	85.7%
HbA1c [%]	11.6 (6.0–15.8)	7 (4.7–13.3)	<0.0001	N/A
Creatinine [mg/dL]	1.1 (0.52–3.23)	0.83 (0.45–3.04)	0.09	-
Urea [mg/dL]	55.5 ± 36.0	52.1 ± 43.7	0.74	-
HDL cholesterol [mg/dL]	31.4 ± 12.6	29 ± 10.0	0.47	-
LDL cholesterol [mg/dL]	85.6 ± 31.8	79.1 ± 34.3	0.46	-
Total cholesterol [mg/dL]	137.9 ± 40	129.2 ± 46.9	0.44	-
Triglycerides [mg/dL]	133.6 ± 59.3	102.0 ± 33.7	0.04	81.4%
Apolipoprotein-E level [mg/dL]	16.1 ± 6.9	15.86 ± 6.6	0.87	-

Note: *p*-values in grey are deemed statistically significant (*p* < 0.05).

**Table 4:** Lipid profiles of survivors versus non-survivors.

Parameter	Survivors (n = 70)	Non-Survivors (n = 10)	*p*-Value	Post-Hoc Statistical Power (α = 0.05, β = 0.2)
Male gender	70.0% (49/70)	50.0% (5/10)	0.36	-
Age [years]	64.4 ± 10.3	63.9 ± 11.1	0.88	-
Rural provenience	61.4% (43/70)	70% (7/10)	0.84	-
Hemoglobin (Hb) [g/dL]	11.4 ± 2.0	9.4 ± 2.3	0.005	74.3%
Hematocrit (Ht) [%]	34.6 ± 5.5	29.3 ± 6.5	0.007	69.0%
HbA1c [%]	8.3 (4.7–14.0)	10 (6.4–16.9)	0.06	-
Creatinine [mg/dL]	0.9 (0.45–3.08)	1.57 (0.90–3.23)	0.0009	N/A
Urea [mg/dL]	48.3 ± 28.2	100.0 ± 62.0	<0.0001	73.9%
HDL cholesterol [mg/dL]	31.7 ± 12.8	25.5 ± 10.1	0.14	-
LDL cholesterol [mg/dL]	84.2 ± 31.9	84.2 ± 36.2	0.99	-
Total cholesterol [mg/dL]	137.1 ± 41.3	129.1 ± 44.4	0.57	-
Triglycerides [mg/dL]	123.9 ± 53.8	148.0 ± 70.5	0.02	17.8%
Apolipoprotein-E level [mg/dL]	15.7 ± 6.8	18.9 ± 6.6	0.17	-
Diabetes duration [%] 5 years 5–10 years 10 years	27.14 35.72 37.14	20.00 30.00 50.00	0.34	-

Note: *p*-values in grey are deemed statistically significant (*p* < 0.05).

## Data Availability

The anonymized patient data used in the elaboration of this study are available upon request from the corresponding authors.
